# Supplementary material for: Stabilizing polymer electrolytes in high-voltage lithium batteries
Source: Nat Commun. 2019 Jul 12;10:3091. doi: 10.1038/s41467-019-11015-0 (PMC6626095; doi:10.1038/s41467-019-11015-0)
Supplement: Supplementary file 1 — Supplementary Information [file 41467_2019_11015_MOESM1_ESM.pdf]

Supplementary Information

Stabilizing Polymer Electrolytes in High-voltage Lithium Batteries

Choudhury et al.

Supplementary Figures

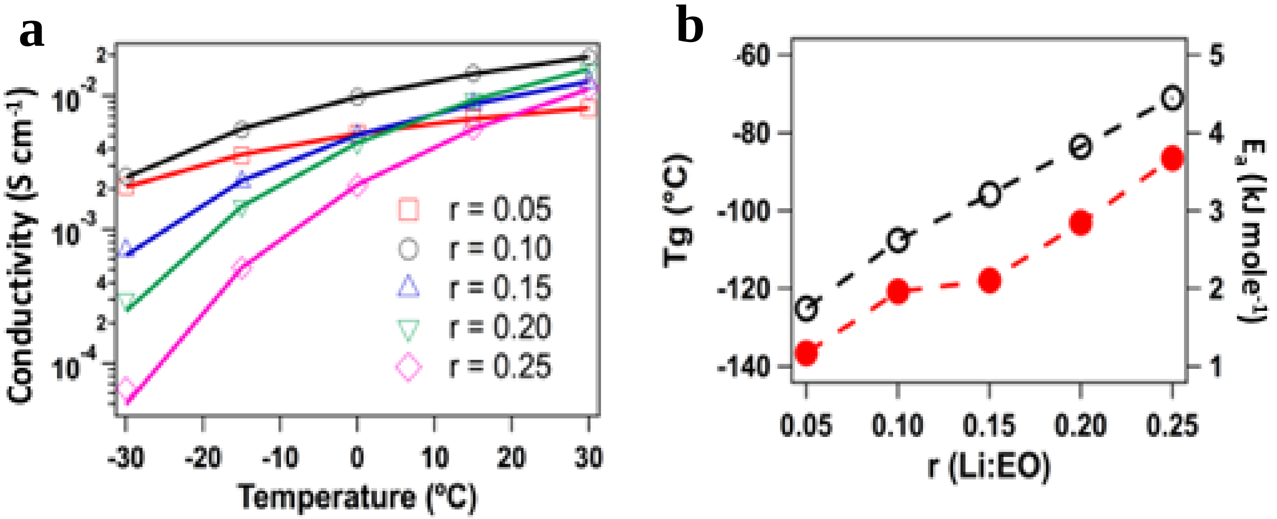

Supplementary Figure 1: Transport and Thermodynamic properties of electrolyte: (a) d.c. conductivity as a function of temperature for different Li:EO (r) ratios. The points represent experimental values and lines are the CFT fitting; **(b)** Variation in glass transition temperature obtained from Differential Scanning Calorimetry measurements in the left axis and Activation Energy obtained from the VFT fitting of conductivity data.

a **b**

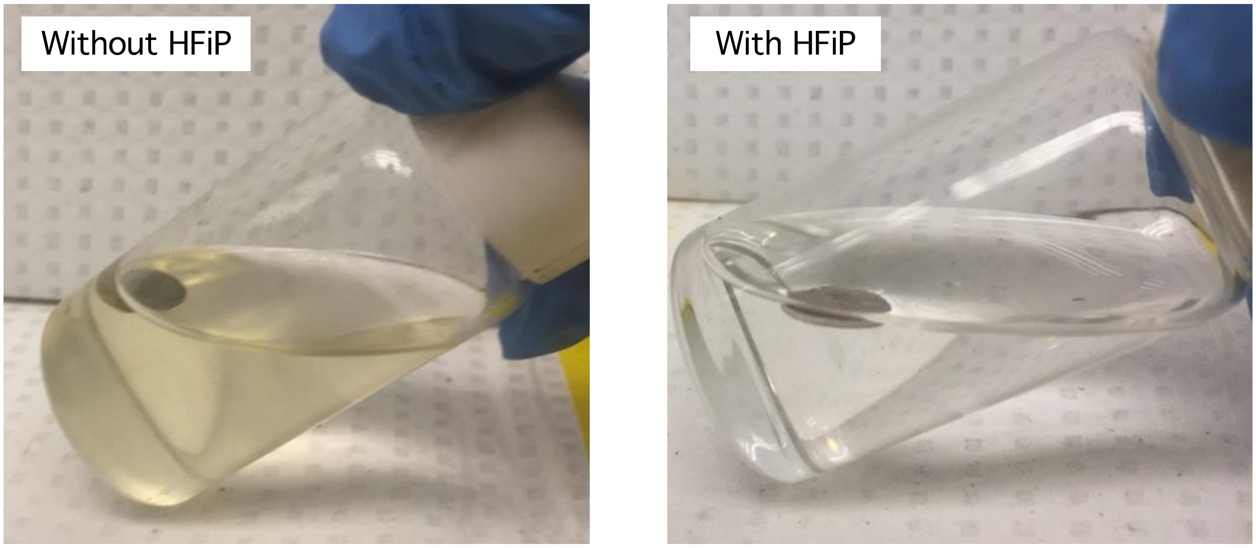

Supplementary Figure 2: Aging behavior of the electrolyte: A piece of lithium metal was added to the diglyme-$LiNO_3$ electrolyte **(a)** without 1wt.% HFiP (left) and **(b)** with 1wt.% HFiP additive (right). The electrolyte solutions were aged for one month in a vial bottle. It is seen that the electrolyte without HFiP additive turns yellow and also the lithium surface becomes blackened, presumably, due to the polymerization of the glyme molecules

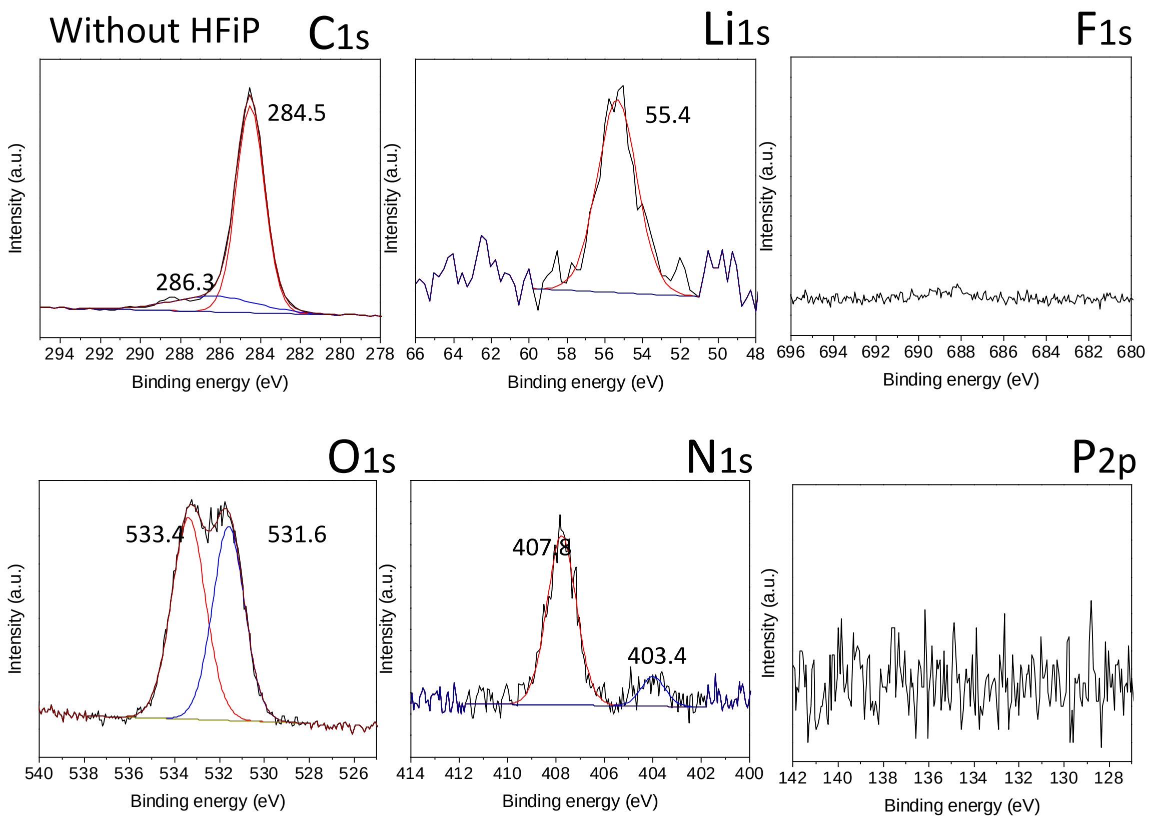

Supplementary Figure 3: Binding energies of different atoms on the surface of lithium obtained from X-ray Photoelectron Spectroscopy. The lithium metal was dipped in an electrolyte solution of diglyme-$LiNO_3$ without any addition of HFIP

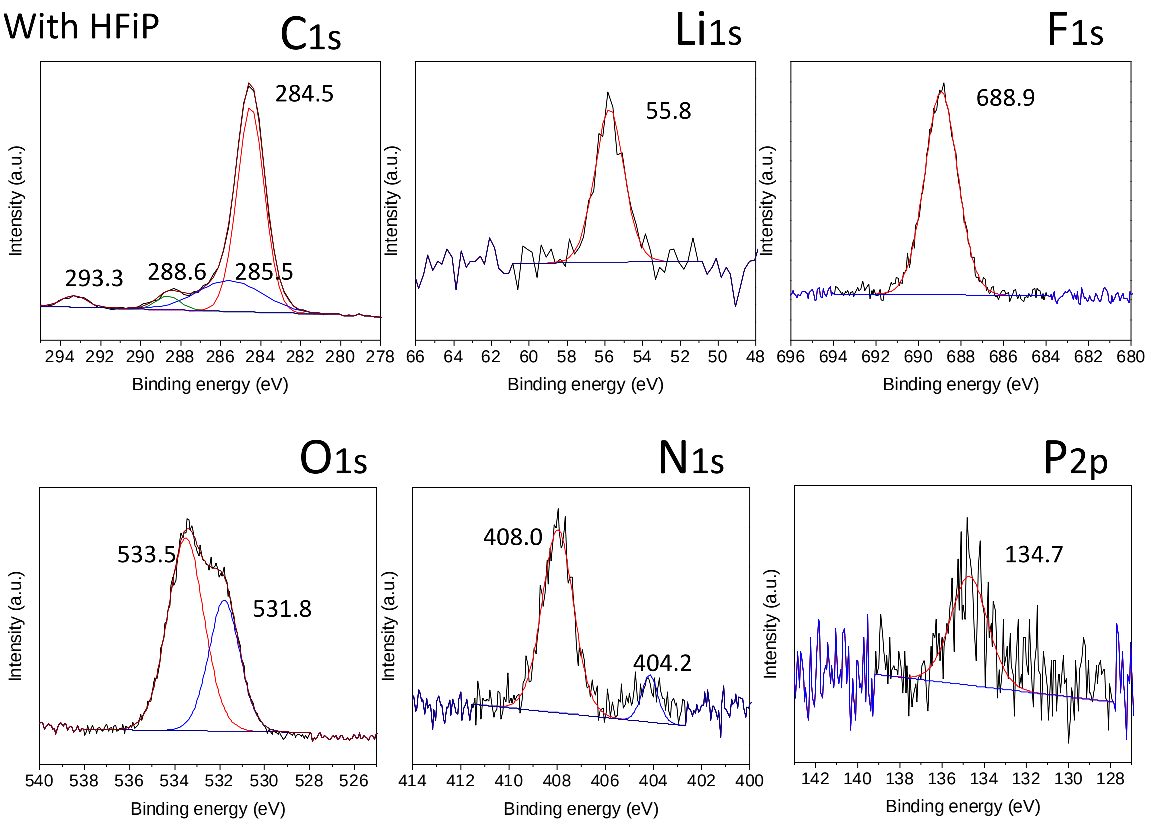

Supplementary Figure 4: Binding energies of different atoms on the surface of lithium obtained from X-ray Photoelectron Spectroscopy. The lithium metal was dipped in an electrolyte solution of diglyme-$LiNO_3$ with 1wt.% HFiP additive

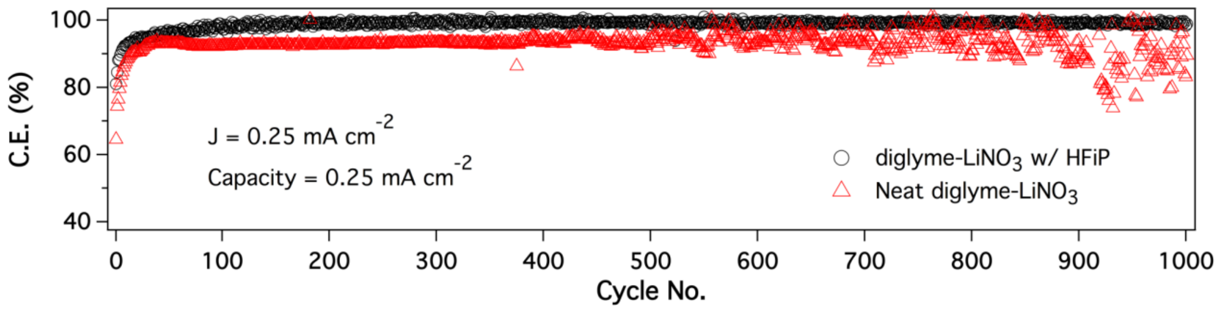

Supplementary Figure 5: Coulombic efficiency measurements in a Li||stainless steel battery at a current density of 0.25mA cm^{-2} and capacity of 0.25mAh cm^{-2}. The black circles represent the diglyme-$LiNO_3$ electrolyte with the HFiP additive and red triangles are for neat electrolyte

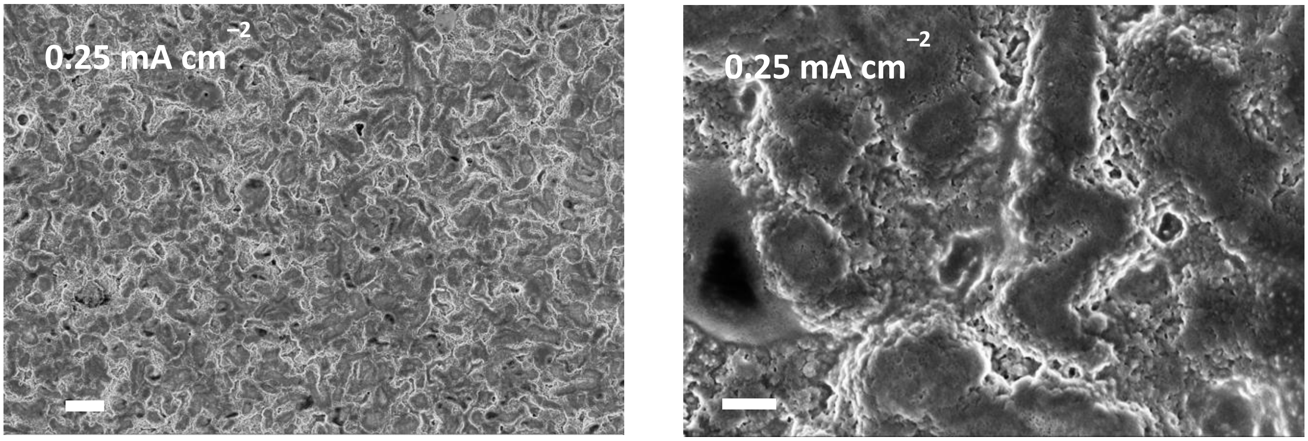

Supplementary Figure 6: Scanning electron microscopy image of stainless-steel substrate after lithium deposition for 24 hours at the current density of 0.25 mA cm^{-2}, using the electrolyte diglyme-$LiNO_3$ with 1wt.% HFiP. The left scale bar represents 10μm and right is 2μm.

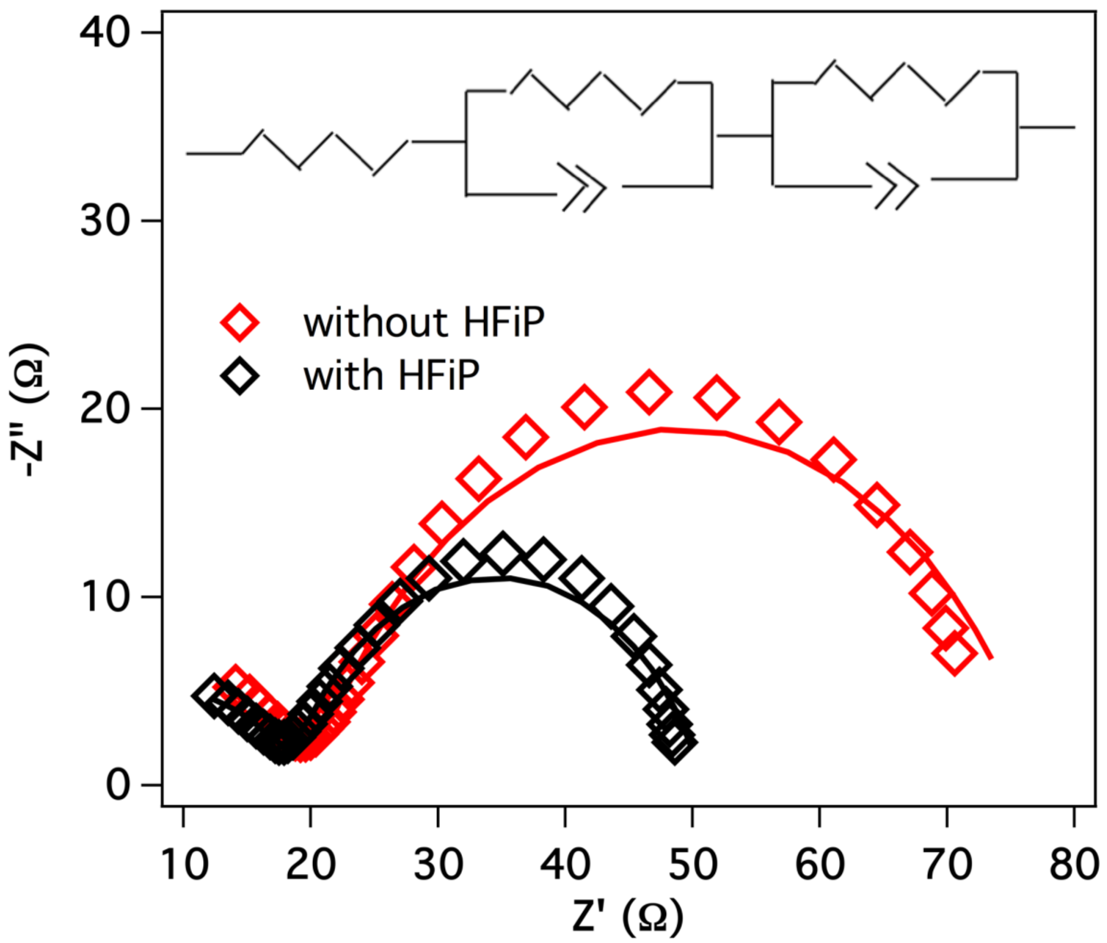

Supplementary Figure 7: Nyquist diagrams obtained from impedance spectroscopy for lithium vs. stainless steel cell that was cycled 100 times at a current density of 1 mA cm^{-2} and capacity of 1 mAh cm^{-2} before depositing lithium onto the stainless-steel electrode. The red symbols are for electrolyte of diglyme-$LiNO_3$; while the black is for the same electrolyte with 1 wt.% HFiP additive. The inset shows the circuit model utilized to fit the data.

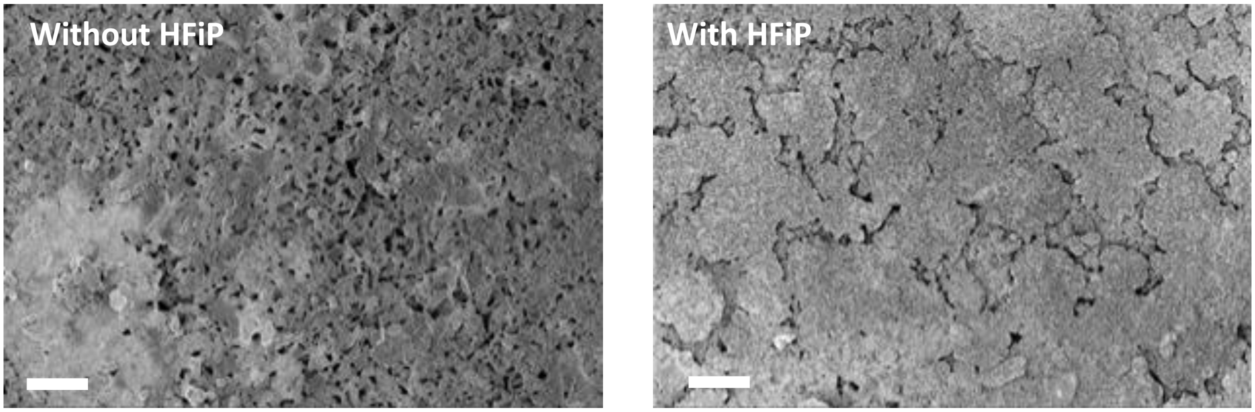

Supplementary Figure 8: Scanning electron microscopy image of stainless-steel substrate after 100 cycles of plating and stripping lithium for 1 hour at the current density of 1 mA cm^{-2}. In the last step lithium metal was plated onto stainless steel electrode. The left image is for the diglyme-$LiNO_3$ electrolyte, without any additive and the right is for same electrolyte with 1wt.% HFiP additive. The scale bar in both images represent 20μm

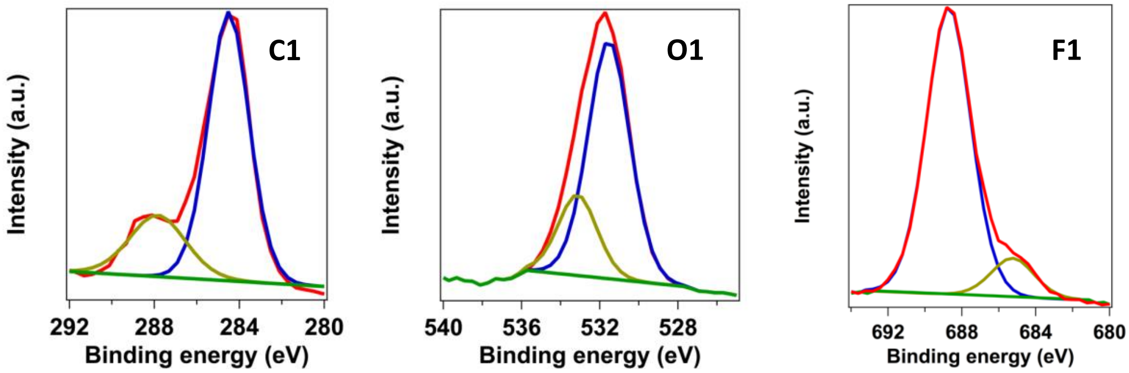

Supplementary Figure 9: Binding energies of different atoms obtained from X-ray Photoelectron Spectroscopy measurements for the lithium surface, extracted from a cell of after cycling 100 times at a current density of 1 mA cm^{-2} and capacity of 1 mAh cm^{-2} in a cell with configuration of Li||stainless steel. electrolyte comprised of diglyme-$LiNO_3$ with 1 wt.% HFiP additive.

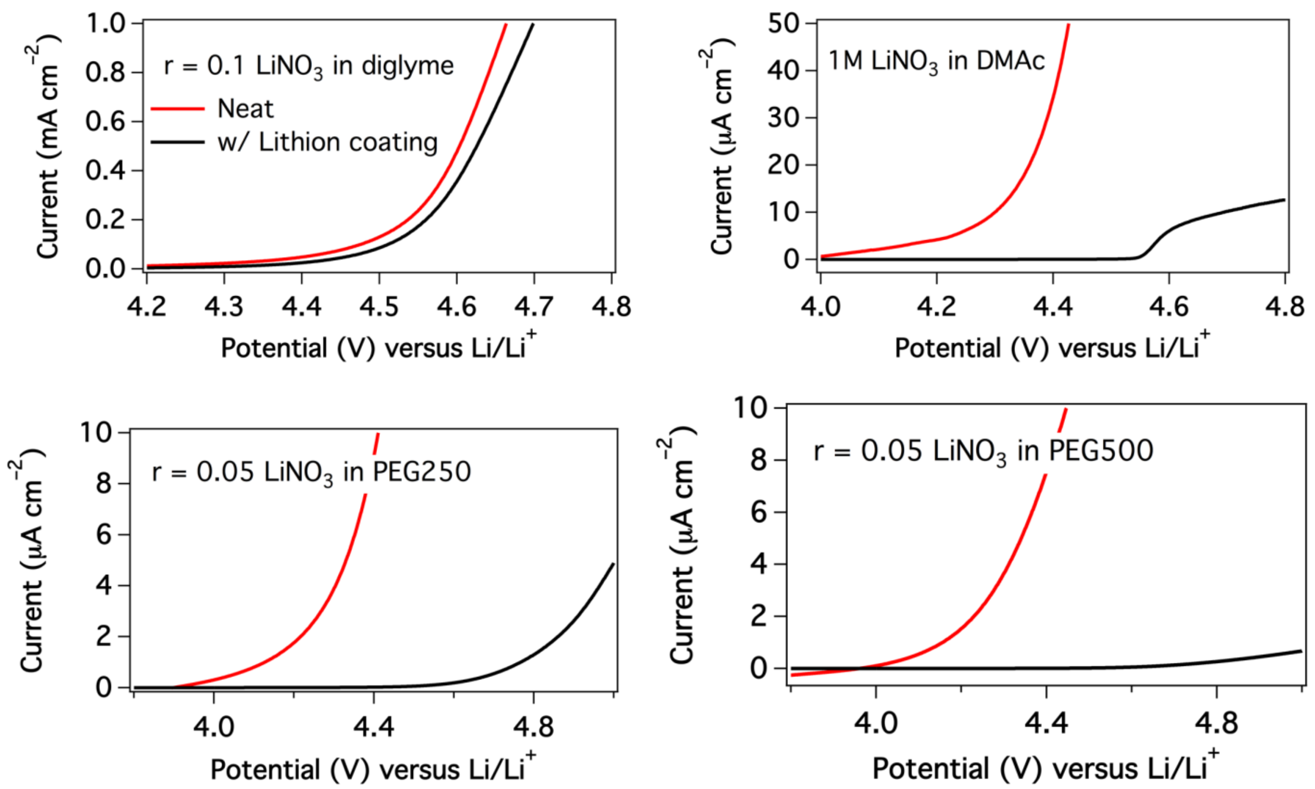

Supplementary Figure 10: Linear scan voltammetry in a three-electrode cell with Ag/AgCl electrode as reference electrodes, while stainless steel used as both reference and counter electrodes. The scan rate utilized was 10mV s^{-1} and the potentials were shifted with reference to Li/Li^{+}. The red curves represent cases where the stainless steel was coated with Lithion layer and black represent pristine stainless-steel electrodes.

Image near the center

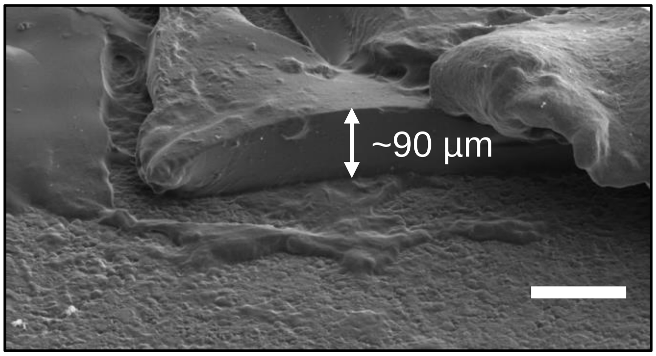

Image near the edge

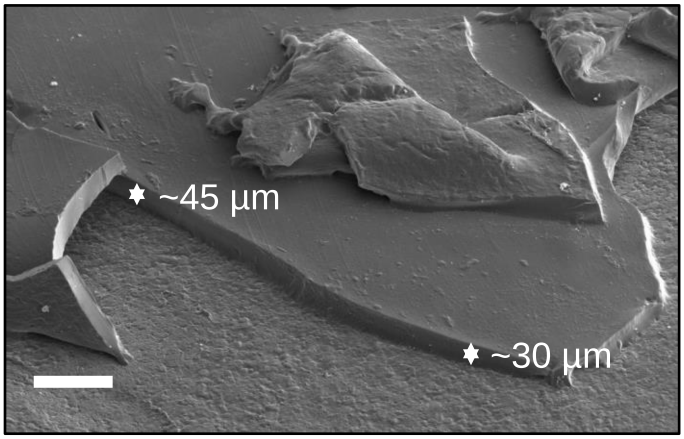

Supplementary Figure 11: Cryo-FIB/SEM images lithion coated NCM surfaces. A lithion layer was present on the NCM cathode and cracked during preparation to reveal its thickness. A thickness gradient was present, from ~90 µm thick near the center to ~30 µm near the edge. The scale bars represent 100µm

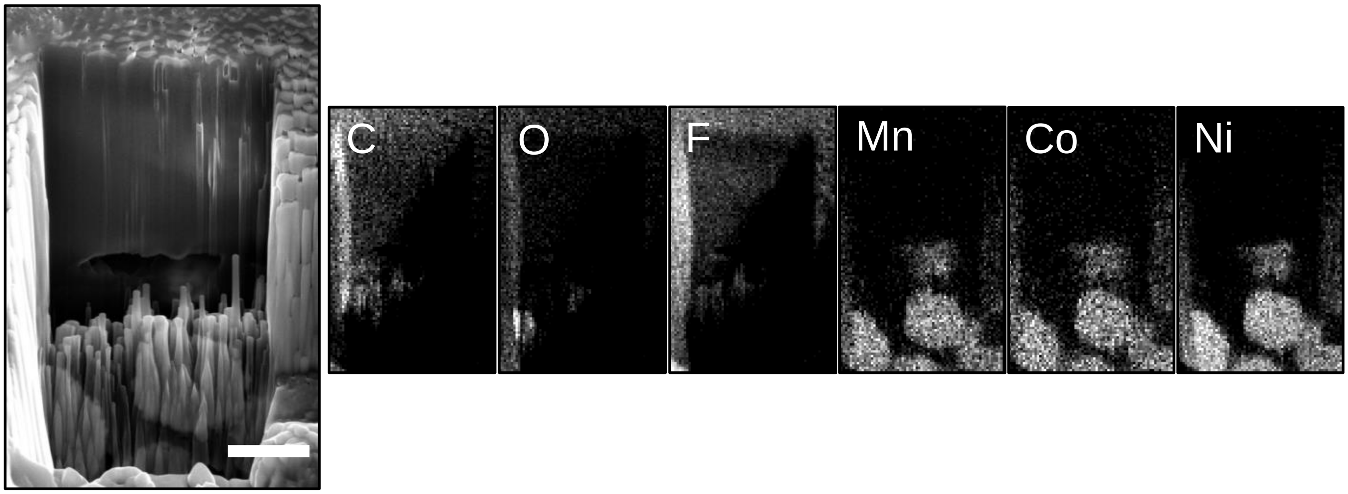

Supplementary Figure 12: Cryo-FIB/SEM images through a thin part of lithion coating NCM surface. Images on the right, show EDX mapping. The scale bar represents 5μm

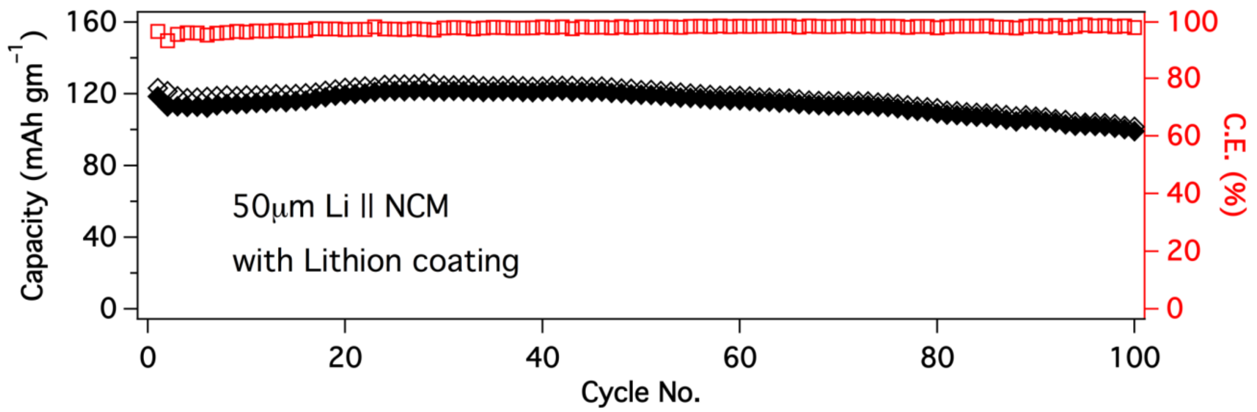

Supplementary Figure 13: Cycling of a thin lithium versus NCM battery, where the capacity of the lithium anode was 10mAh cm^{-2} and that of cathode was 2mAh cm^{-2}. Here, the NCM cathode was coated with a layer of Lithion layer. The current density is 0.4mA cm^{-2} (C/5)

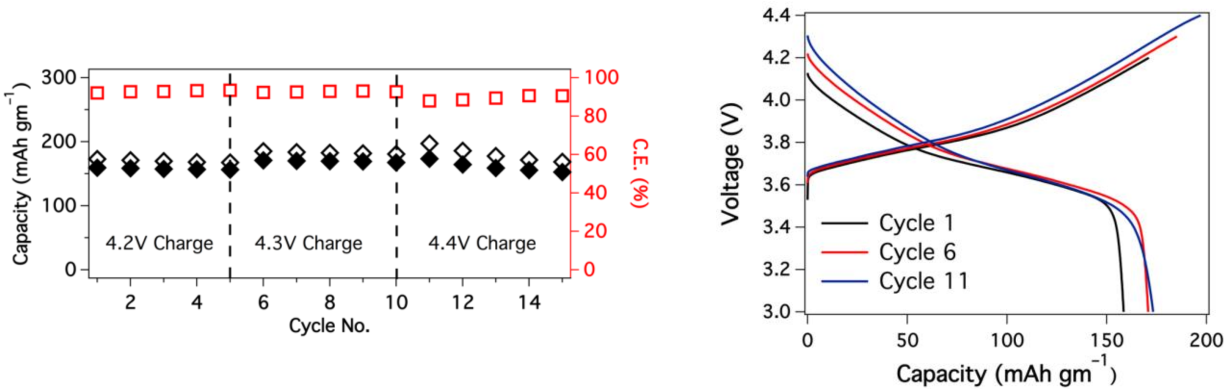

Supplementary Figure 14: Cycling of a thin lithium versus NCM battery, where the capacity of the lithium anode was 10mAh cm^{-2} and that of cathode was 2mAh cm^{-2}. Here, the NCM cathode was coated with a layer of Lithion layer. The current density is 0.2mA cm^{-2} (C/10)

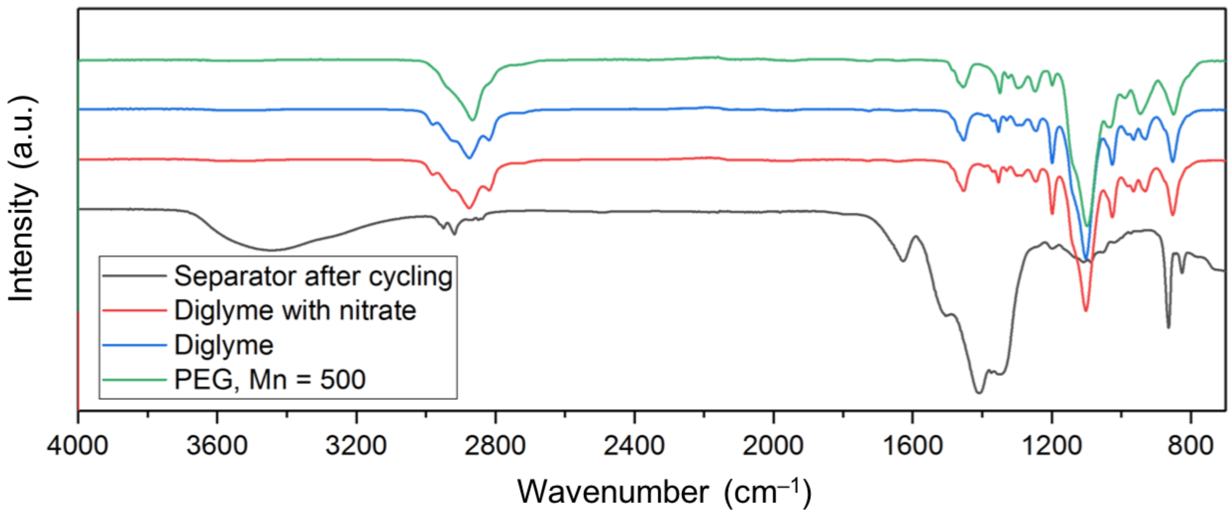

Supplementary Figure 15: FTIR Spectra for diglyme electrolyte infused separator extracted from a Li||NCM cell without any Lithon coating, after it was cycled twice at C/10. It is compared with neat diglyme solvent, diglyme with $LiNO_3$ and PEG500 liquid.

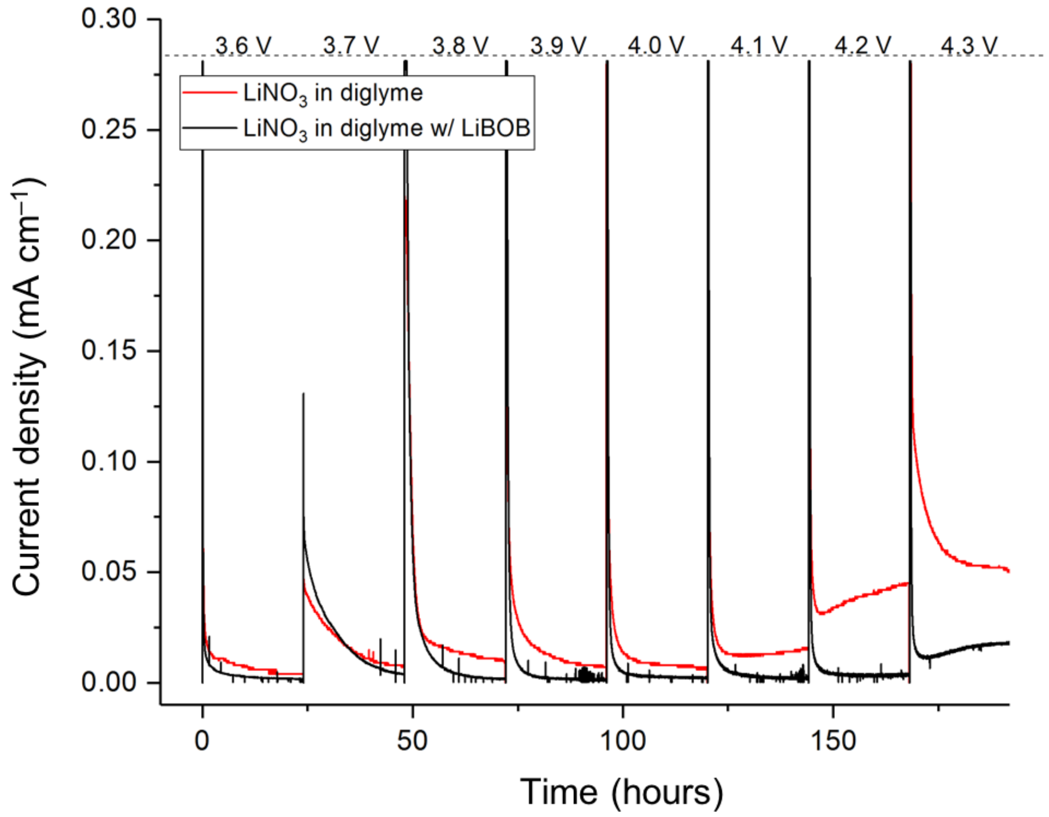

Supplementary Figure 16: Floating experiments using Li||NCM cell, where the cells are charged at different voltages for 24 hours each and the leak current is recorded to determine the side reactions at the operated voltages. The red line is for the diglyme-$LiNO_3$-HFiP electrolyte, while the black line is result for the same electrolyte with 0.4M LiBOB salt as additive

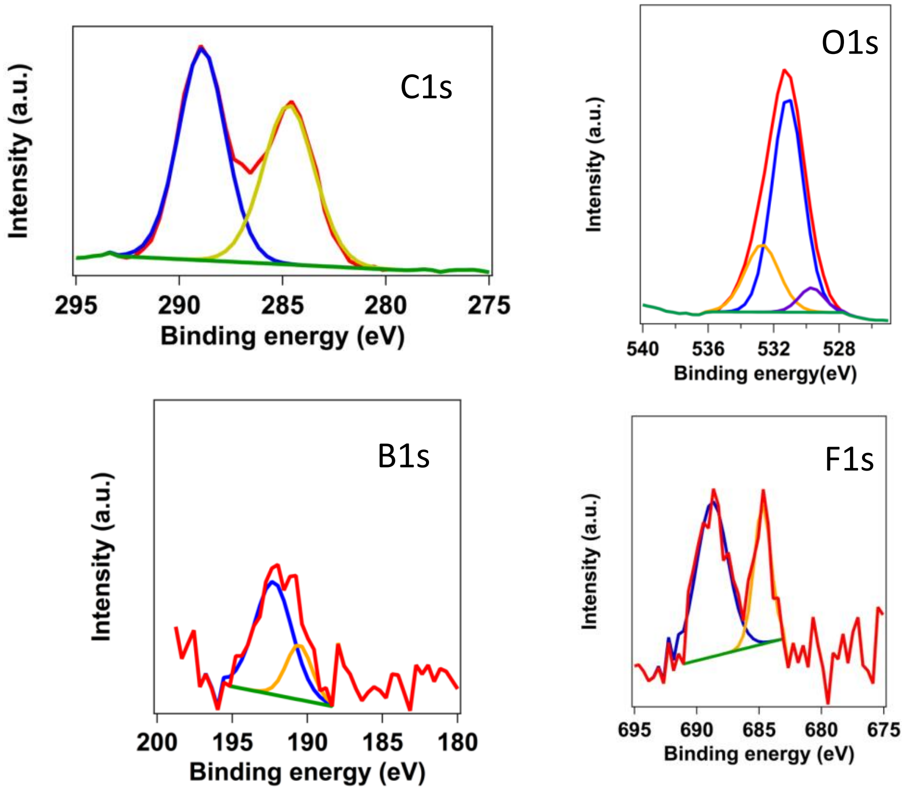

Supplementary Figure 17: XPS spectra from obtained from the surface of lithium metal anode harvested from a Li||NCM cell cycled twice at a C/10 rate using the electrolyte diglyme-$LiNO_3$-HFiP with 0.4M LiBOB additive. Here, the Fluorine spectra indicates that there is presence of both LiF and -CF3 content from the HFiP, while the LiBOB plays a role of forming boro-oxolate compounds in the anodic interfacial layer due to low potential reduction

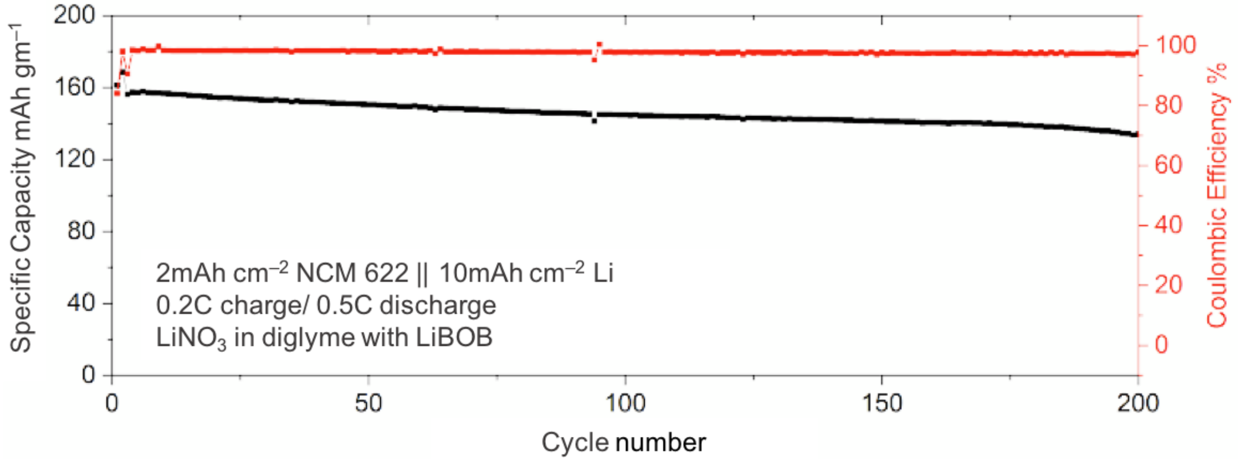

Supplementary Figure 18: Li||NCM cycling results using a rate of 0.2C charge and 0.5C discharge. The cathode loading is 2mAh cm^{-2} and the lithium metal anode is 50μm thick that corresponds to 10mAh cm^{-2} capacity. The electrolyte used here is diglyme-$LiNO_3$-HFiP with 0.4M LiBOB salt additive.

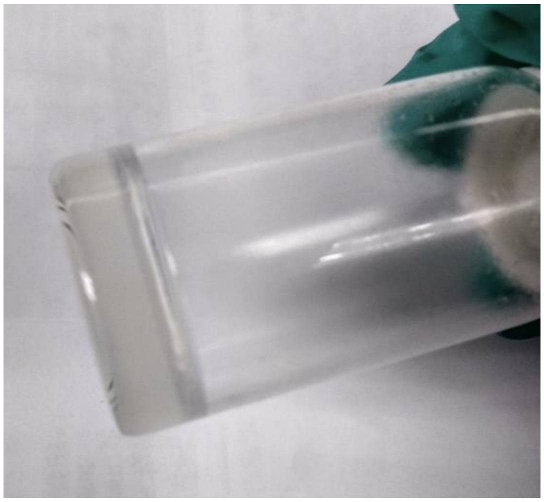

Supplementary Figure 19: Image showing the gel electrolyte used for cycling at room temperature. The composition is 1wt.% 100k PEG in diglyme with $LiNO_3$ and HFiP.

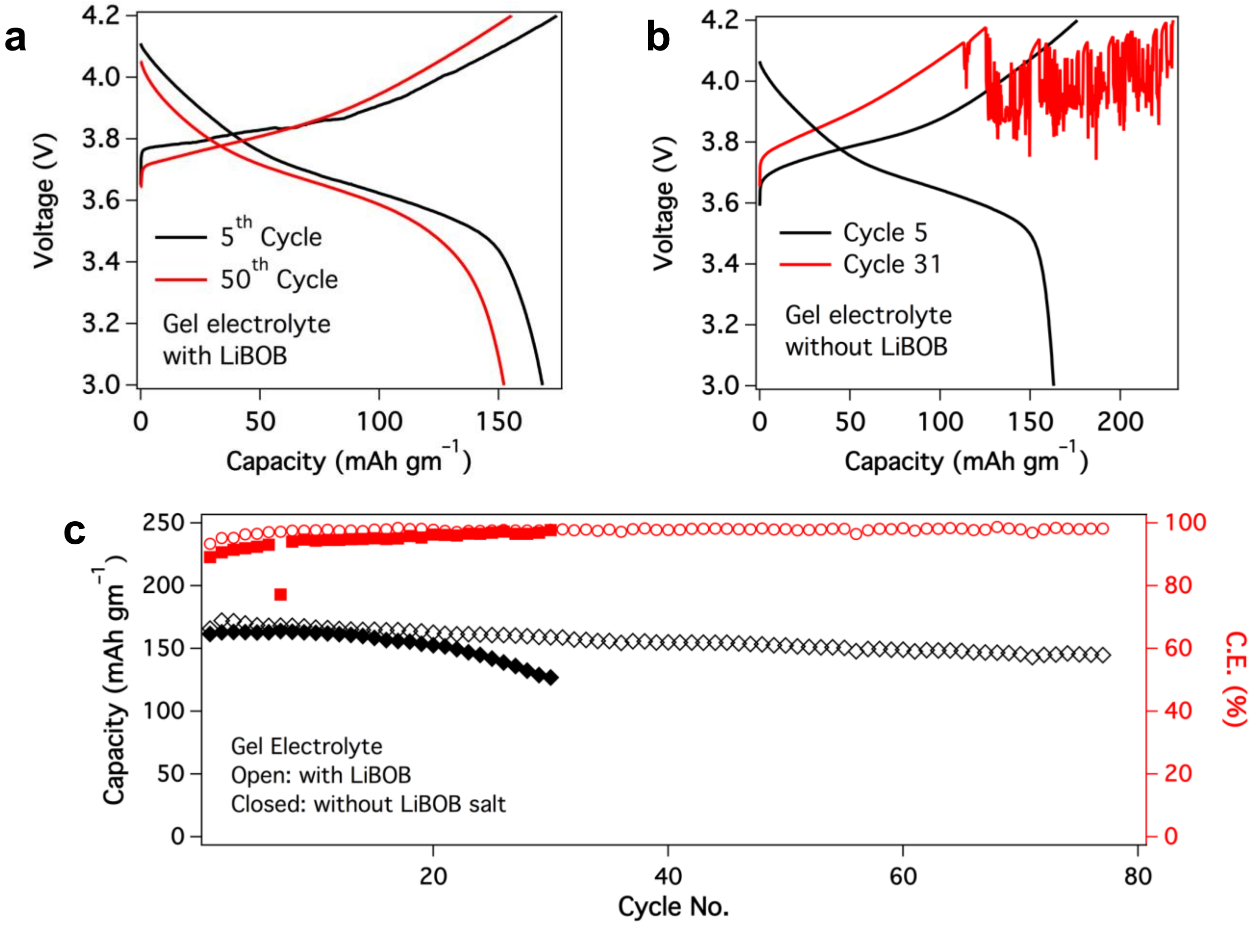

Supplementary Figure 20: Voltage profile of Li||NCM cell with a thin (50µm) lithium and 2mAh cm^{-2} cathode cycled at C/5 rate using the gel electrolyte comprising of 1 wt.% PEG 100k in diglyme-$LiNO_3$-HFiP, **(a)** with LiBOB salt additive and **(b)** without LiBOB. (c) Cycling performance of the gel electrolyte with (open symbols) and without LiBOB salt (closed symbols).

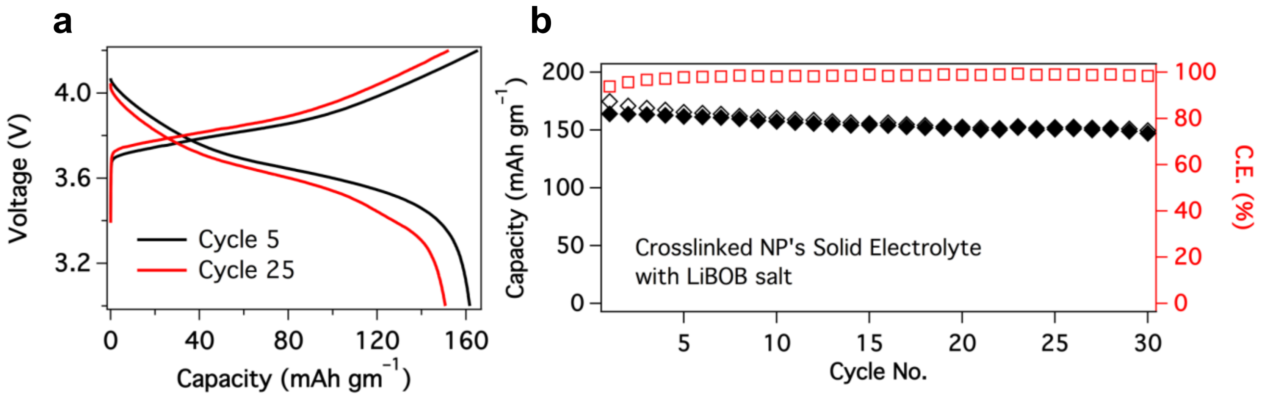

Supplementary Figure 21: Electrochemical characterization: (a) Voltage profile of Li||NCM cell with a thin (50μm) lithium and 2mAh cm^{-2} cathode cycled at C/10 rate using crosslinked hairy nanoparticles soaked with the electrolyte diglyme-$LiNO_3$-HFiP and LiBOB additive, **(b)** Cycling profile showing the coulombic efficiency and charge/discharge capacity

Degradation products of diglyme at high voltages.

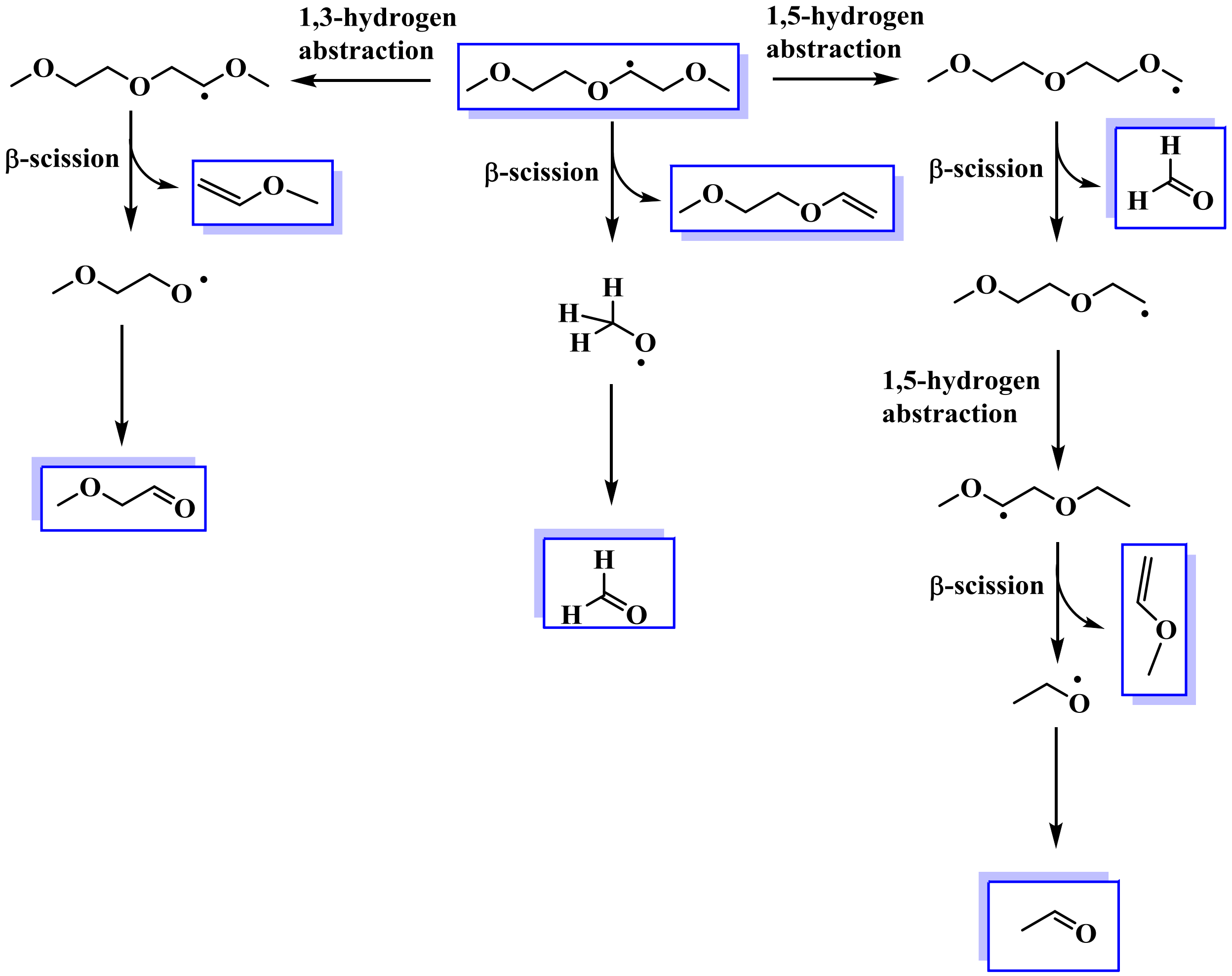

Supplementary Figure 22. Possible degradation mechanisms for the initially formed diglyme at high voltage. The new peaks found in the FTIR spectrum of diglyme at high voltage can be assigned to the aldehydes and monosubstituted alkenes shown here.

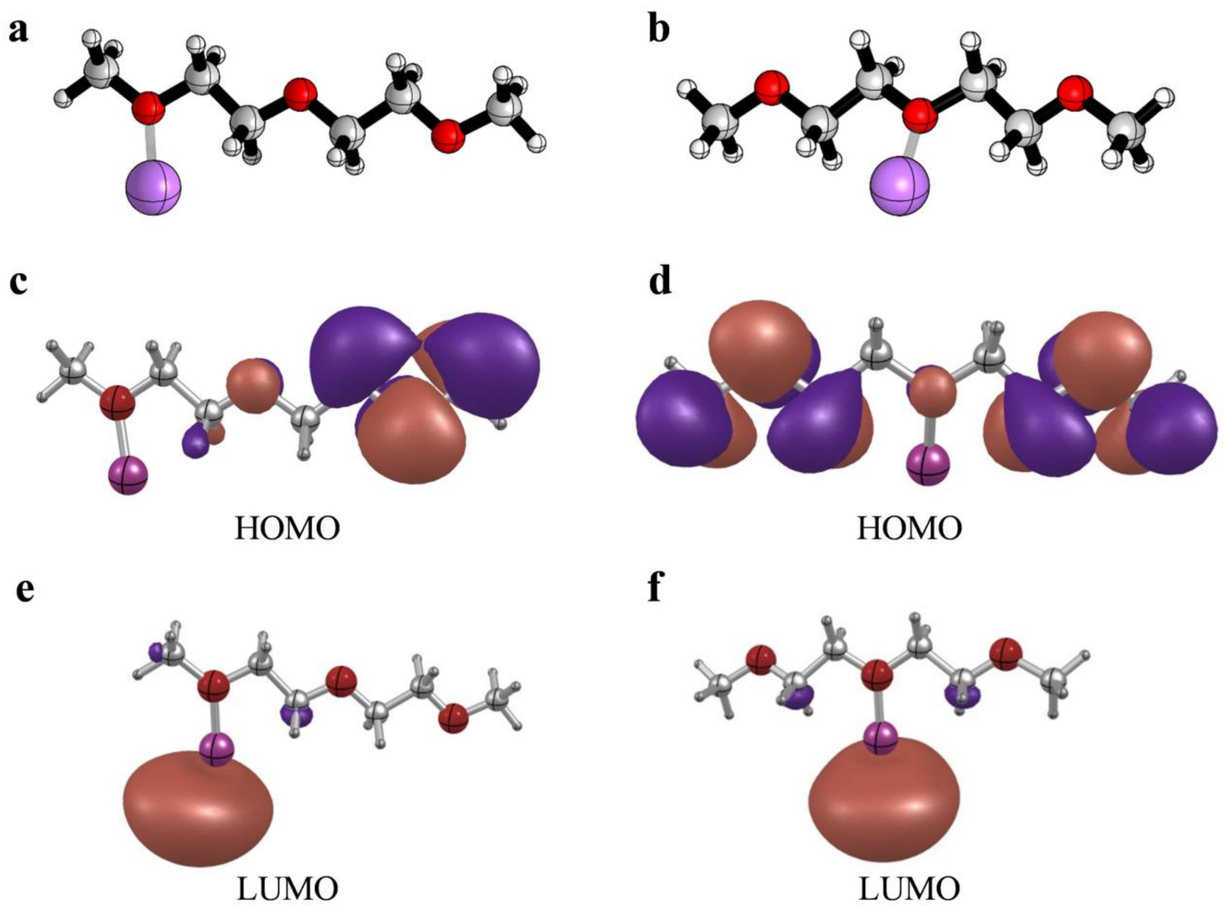

Supplementary Figure 23. Optimized geometries and calculated frontier molecular orbitals of diglyme☐☐☐Li^+ complexes.

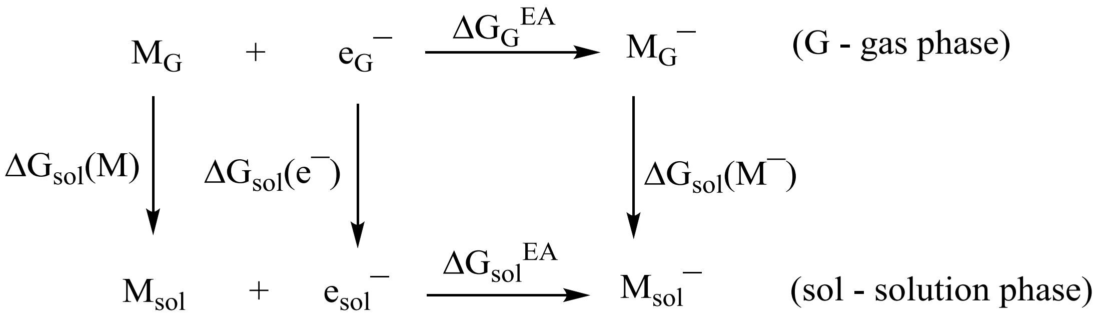

Supplementary Figure 24. Thermodynamic cycle used to calculate the oxidation/reduction potential of diglyme and its oligomers formed via reactions with BOB.

Supplementary Table1. Calculated frontier molecular orbital energies and HOMO-LUMO energy gaps in diglyme and its complexes with Li^+.

**System**	E(HOMO), eV	E(LUMO), eV	E(HOMO–LUMO), eV
Diglyme	−9.3	3.4	12.7
Diglyme□□□Li^+, Figure 23 (a)	−9.7	0.6	10.3
Diglyme□□□Li^+, Figure 23 (b)	−10.1	0.6	10.7

Supplementary Methods

Computational Details

The structures of diglyme, BOB, and oligomers are optimized in the gas-phase at hybrid density functional theory (DFT) level by employing the wB97X-D functional.[1,2] This functional includes effects due to long-range interaction and dispersion, and has been successful in accurately modeling the geometries, thermochemistry, and electronic properties of a variety of molecular systems. We have employed the double zeta 6-311G(d,p)[3] basis sets implemented in the Gaussian suite of programs for all calculations.[4] Vibrational frequencies are computed at the same level of theory to ensure that the optimized geometry represents a true minimum. Further, single point calculations are performed on these structures by using a polarizable continuum model to mimic the effects of diglyme.[5] In order to find the Gibb's free energy changes due to the addition or removal of an electron to a neutral molecule or an anion, we used an electron solvation free energy of 1.63 eV as determined from previous studies.[6] The redox potential of diglyme and its oligomers with BOB are calculated by using the thermodynamic cycle presented in the Supplementary Scheme 1.[7]

By using the thermodynamic cycle shown in Supplementary Figure 24, Gibbs free energy change in solution phase (${\Delta G_{sol}}^{EA}$) during oxidation process could be estimated from supplementary equation (1).

$$ {\Delta G_{sol}}^{EA} = {\Delta G_G}^{EA} + \Delta G_{sol}(M^-) - \Delta G_{sol}(M) - \Delta G_{sol}(e^-). \quad (1) $$

Then, the oxidation potential for a given molecule (M) is calculated as

$$ E_0 = -{\Delta G_{sol}}^{EA}/F. \quad (2) $$

where F is the Faraday constant.

Practically, it is difficult to calculate the solvation free energy of an electron, $\Delta G_{sol}(e^-)$. Therefore, we have calculated the relative oxidation potential with respect to Li/Li^+ electrodes (-3.04 eV) using supplementary equation (3).

$$E^0 = -(\Delta G_G{}^{EA} + \Delta G_{sol}(M^-) - \Delta G_{sol}(M))/F + 3.04. \quad (3)$$

Supplementary References

1. Chai, J.-D. & Head-Gordon, M. Long-range corrected hybrid density functionals with damped atom–atom dispersion corrections. *Phys. Chem. Chem. Phys.* **10**, 6615–6620 (2008).
2. Chai, J.-D. & Head-Gordon, M. Systematic optimization of long-range corrected hybrid density functionals. *J. Chem. Phys.* **128**, 84106 (2008).
3. Hariharan, P. C. & Pople, J. A. The influence of polarization functions on molecular orbital hydrogenation energies. *Theor. Chim. Acta* **28**, 213–222 (1973).
4. M. J. Frisch, et. al, gaussian 09, Revision C.01, Gaussian, Inc., Wallingford, CT, 2009.
5. Tomasi, J., Mennucci, B. & Cammi, R. Quantum Mechanical Continuum Solvation Models. *Chem. Rev.* **105**, 2999–3094 (2005).
6. Zhan, C.-G. & Dixon, D. A. The Nature and Absolute Hydration Free Energy of the Solvated Electron in Water. *J. Phys. Chem. B* **107**, 4403–4417 (2003).
7. Roy, L. E.; Jakubikova, E.; Guthrie, M. G.; Batista, E. R. Calculation of One-Electron Redox Potentials Revisited. Is It Possible to Calculate Accurate Potentials with Density Functional Methods? *J. Phys. Chem. A*, **113**, 6745–6750 (2009).
